# A Porphyrin-DNA Chiroptical Molecular Ruler With Base Pair Resolution

**DOI:** 10.3389/fchem.2020.00113

**Published:** 2020-02-26

**Authors:** Jonathan R. Burns, James W. Wood, Eugen Stulz

**Affiliations:** ^1^Department of Chemistry, University College London, London, United Kingdom; ^2^School of Chemistry & Institute for Life Sciences, University of Southampton, Southampton, United Kingdom

**Keywords:** DNA, porphyrin, FRET, CD spectroscopy, ethidium bromide intercalation, exciton coupling

## Abstract

DNA-based molecular rulers enable scientists to determine important parameters across biology, from the measurement of protein binding interactions, to the study of membrane dynamics in cells. However, existing rulers can suffer from poor nanometre resolution due to the flexible nature of linkers used to tether to the DNA framework. We aimed to overcome this problem using zinc and free-base porphyrin chromophores attached via short and rigid acetylene linkers. This connectivity enables the distance and angle between the porphyrins to be fine-tuned along the DNA scaffold. The porphyrins undergo favorable energy transfer and chiral exciton coupling interactions to act as highly sensitive molecular ruler probes. To validate the system, we monitored the detection of small changes in DNA structure upon intercalation of ethidium bromide. CD spectroscopy showed the porphyrins undergo highly sensitive changes in excitation coupling to facilitate base pair resolution of the novel system.

## Introduction

Förster resonance energy transfer (FRET) is routinely used in the determination of conformational changes or intermolecular interactions in biomolecules, where it gives insight into function and activity. However, most FRET systems have chromophores attached through long and flexible linkers which can limit their sensitivity (Wang et al., 2003; Sabanayagam et al., 2005; Preus and Wilhelmsson, 2012). Since the interaction of chromophores, in view of energy transfer, is highly dependent on their alignment, and FRET has an inverse sixth order distance (R) dependency, this does not allow for a precise distance measurement with sub-nanometre resolution due to fluctuations in position of the FRET pair (Wang et al., 2003), which can be overcome to some extent using time resolved FRET (trFRET) (Klostermeier and Millar, 2001). In addition, when the FRET pair distance is comparable to the linker length, contact quenching can occur. Yet the detection of small structural changes in DNA is highly desirable as it can give information on the DNA topology, e.g., upon binding of proteins (Andrabi et al., 2014), in intercalator–DNA interactions (Biebricher et al., 2015), or in base pair mismatches (Rossetti et al., 2015). To this end, tailor made DNA multi-chromophore systems are now well-established (Malinovskii et al., 2010; Teo and Kool, 2012). Specific systems include, for example, Cy3 and Cy5 dyes which are typically attached through short alkynyl linkers to the nucleobase and can provide useful FRET analysis at short distances, though this was not used for analysis of conformational changes (Hall et al., 2012). Analogously, fluorescent base analogs which were incorporated within the base stacking region of the DNA and thus are inherently held rigidly in place, resulted in a high control of the orientation factor (κ) and hence very distinct FRET changes as the number of bases separating the base analogs were varied (Borjesson et al., 2009). The combination of Cy5/Cy3, however, is suitable to detect global structural changes in hairpin ribozymes (Bates et al., 2005), and in bivalent peptide complexes (Eberhard et al., 2011), even when having flexible linkers. Gating of the Cy5 dye with a green laser enabled FRET values at much shorter distances to be obtained than in conventional systems (Bates et al., 2005). Stilbene chromophores, which were attached to both ends of a DNA hairpin, showed exciton-coupled circular dichroism (EC-CD) which varied strongly along a helical turn and could serve as a molecular ruler; however, the CD signatures are very complex and not straight-forward to interpret (Lewis et al., 2005). The use of pyrenes has shown strong fluorescence enhancement when attached to DNA with short linkers and can serve as a structure-sensitive probe for DNA (Mayer-Enthart and Wagenknecht, 2006). Ultrafast Energy Transfer in pyrene dimers yielded information on structural dynamics of DNA, but the system is complicated by the presence of two electronic coupling pathways, involving the base pair of the DNA (Trifonov et al., 2005).

On a single molecule level, total internal reflection fluorescence (TIRF) microscopy, combined with single molecule FRET (TIRF-smFRET) enables single base pair resolution with 100 ms temporal resolution, though this requires sophisticated equipment setup (Holden et al., 2010). DNA origami tiles were used as an elegant breadboard for measuring distances using single molecule FRET systems (Steinhauer et al., 2009; Stein et al., 2011), but at much larger distances than single base pair resolution. Gold nanoparticles (AuNPs) have also been investigated and held at specific distances using dsDNA spacers; the plasmon coupling of 80 nm AuNPs was shown to allow distance measurements between 1 and 80 nm with a time resolution <50 ms and with absolute distance errors ranging from <1 nm to around 20 nm (Reinhard et al., 2005). Plasmon rulers could certainly be an alternative to FRET for *in vitro* single-molecule experiments (Sonnichsen et al., 2005), in particular because sub-nanometre resolution can be achieved (Liu et al., 2006). Methods other than FRET or plasmon resonance in using DNA as a molecular ruler that have been used include pulsed electron paramagnetic resonance (PELDOR) spectroscopy (Schiemann et al., 2004), X-ray scattering (Mathew-Fenn et al., 2008), and EPR spectroscopy (Nguyen et al., 2014).

Despite the advances made in determining the impact of sequence and external factors on the structure of DNA, including in a time dependent manner, a system with a simple optical readout that gives unambiguous information on local changes down to the single base pair level is still missing. We have studied the characteristics of porphyrin modified DNA extensively (Fendt et al., 2007). Notably a mixed free-base and zinc porphyrin zipper-array allowed for reversible formation of photonic wires (Nguyen et al., 2009). In particular CD spectroscopy has proven to be an invaluable tool in analyzing interactions in porphyrin-DNA due to the strong exciton coupling between the porphyrins, which shows strong dependence on the type of linker between porphyrin and DNA used, as well as on the underlying sequence of the DNA (Brewer et al., 2011; Singleton et al., 2016). Thus this system seemed perfectly well-suited to investigate its ability to monitor the local changes in DNA structure.

## Experimental

The synthesis of the porphyrin-dU building block and its incorporation into DNA were performed as described previously (Fendt et al., 2007). Phosphoramidite chemistry and solid support DNA synthesis followed standard protocols for the natural nucleotides. For the incorporation of the porphyrin-dU, the phosphoramidite was dissolved in MeCN-DCM 10:1, and an extended coupling time of 5 min was used. The DNA strands were purified and analyzed using RP-HPLC. Post-synthetic metalation of the porphyrin strand was done in an aqueous solution using Zn(OAc)_2_ as published earlier (Brewer et al., 2011).

UV-Vis spectroscopy was conducted using a Varian Cary 300 Bio spectrometer with quartz cells of 1 cm path length; scans were carried out at 25°C covering 200–800 nm. Fluorescence spectroscopy was conducted using a Varian Cary Eclipse spectrometer with quartz cells of 1 cm path length; scans were carried out at 25°C with excitation wavelength at λ = 425 nm. CD spectra were recorded at beamline B23 (Module B) at Diamond Light Source, equipped with an Olis DSM20 Monochromator and a photo multiplier tube detector. CD titration with ethidium bromide (EtBr) was performed using a 1.5 mM stock solution to give a total volume 500 μL (4 μM DNA), and a concentration of EtBr equal to 40, 80, 200, 400, and 800 μM. Concentrations and buffers are given in the figure legends. The theoretical predictions of FRET were performed as described previously, using a custom build MATLAB based program FRETmatrix described elsewhere (Preus et al., 2013). The geometry of the porphyrin-DNA duplexes was modeled using Schrödinger's software MacroModel (Mohamadi et al., 1990).

The data for the titration were analyzed using a non-linear fit of the Scatchard's plot of *r*/*C*_f_ vs. *r* using the following equation for n binding sites (Vardevanyan et al., 2003; Minasyan et al., 2006):

(1) $$\begin{array}{l} {r/C}_{f}=K\left(1-nr\right){\left[\frac{1-nr}{1-\left(n-1\right)r}\right]}^{n-1} \end{array}$$

where *r* = *C*_b_/*C*_p_, *C*_b_ = [EtBr]_bound_, *C*_b_ = *C*_0_ – *C*_f_, *C*_0_ = [EtBr]_total_, *C*_f_ = [EtBr]_unbound_, *C*_p_ = [phosphate groups].

For **Y1**, the Δθ at 415 nm was used, and for **Y2** the Δθ at 420 nm was used. The values of |θ_max_ – θ_min_| were fitted to obtain the theoretical value of maximum change (highest possible intercalation. From this, the fraction of bound EtBr was calculated, assuming that the intercalation on a global scale leads to a linear response of Δθ.

## Results and Discussion

### Synthesis and Stability of the FRET System

**Figure 1:**
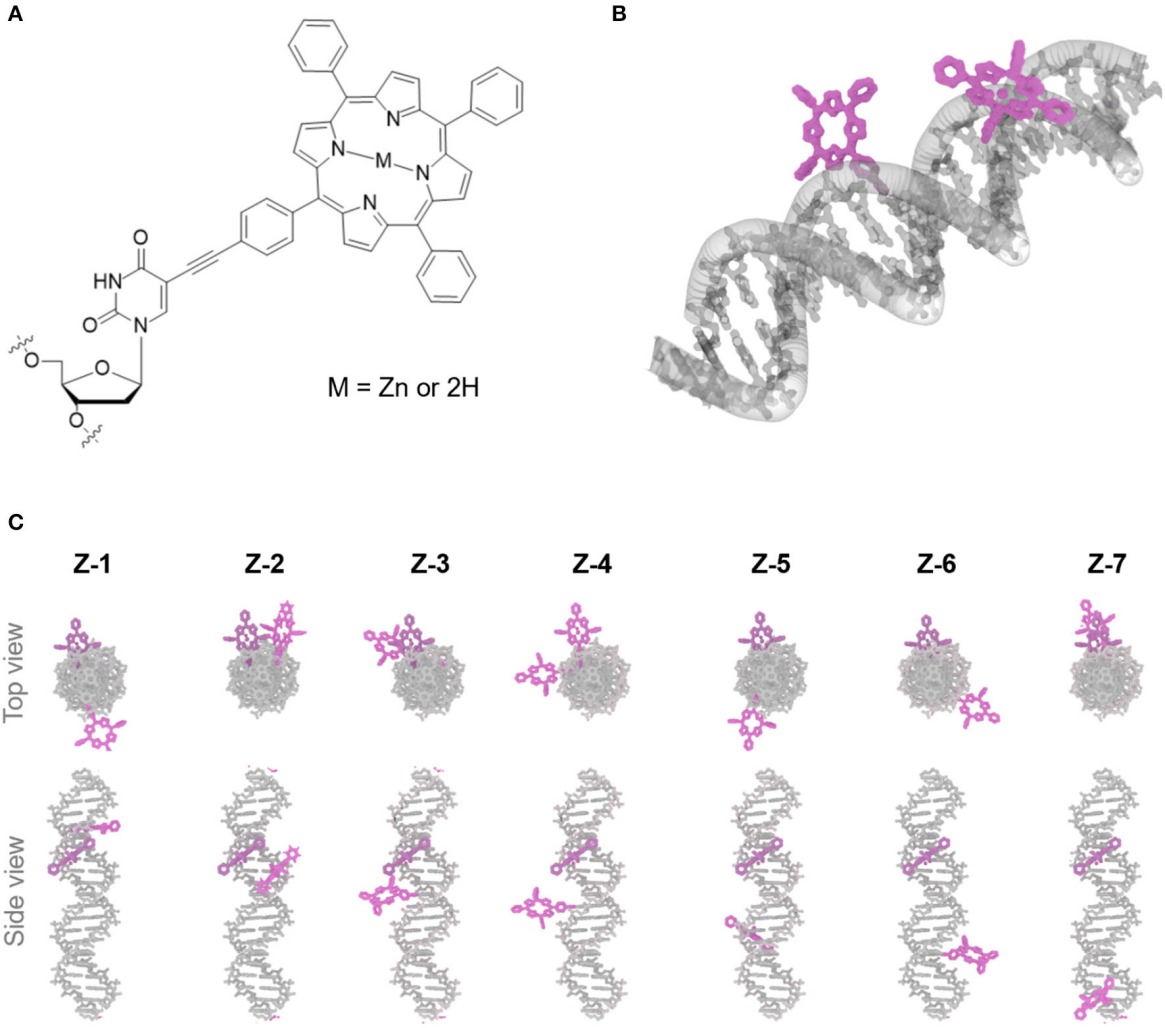
**(A)** Chemical structure of porphyrin DNA modification connected *via* a rigid acetylene linker, the central cavity contains either a Zn or free-base (2H). **(B)** 3D rending of duplex DNA (gray) modified with two porphyrin molecules (purple). **(C)** Top and side view of porphyrin-DNA duplexes assayed containing Zn-porphyrin (donor) and 2H-porphyrins (acceptor) (purple), the Zn-porphyrin position is held constant, whilst the 2H-porphyrin is varied, giving rise to seven different porphyrin-porphyrin distances and angles.

**Figure 2:**
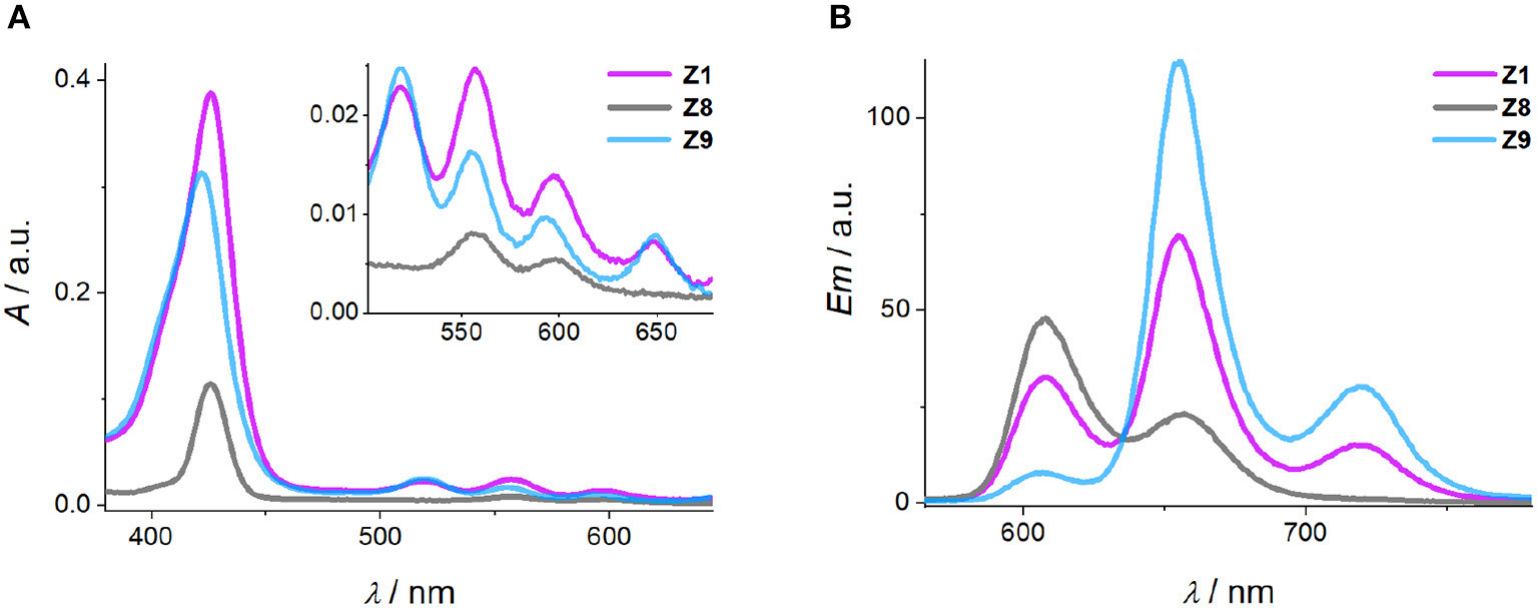
Representative absorbance **(A)** and emission **(B)** spectra of the Zn and 2H-porphyrin FRET pair (**Z1**), Zn-porphyrin (**Z8**) and 2H-porphyrin (**Z9**). [DNA] = 2 μM, 100 mM NaCl, 100 mM Na_2_HPO_4_ buffer pH 7.0.

In this study, we used an electronically coupled system based on zinc porphyrin as donor (**Zn-P**) and free-base porphyrin as acceptor (**2H-P**) which has previously shown efficient energy transfer within a DNA supramolecular assembly (Bouamaied et al., 2007; Nguyen et al., 2009; Brewer et al., 2011). We built an array of DNA duplexes to study the energy transfer properties for this two-porphyrin system (see Figure 1, **Z1** to **Z7**, and ESI for sequences). The position of the Zn-P was fixed throughout, whilst the 2H-P was varied. The corresponding base pair separation between the porphyrins change both the distance and dipole-dipole alignment between them. The porphyrin-nucleoside building block was synthesized according to previously published procedures and used for solid phase synthesis of the porphyrin-DNA (Bouamaied et al., 2007; Brewer et al., 2011). The thermal UV-denaturing analysis shows a *T*_m_ of ~47°C for all porphyrin containing duplexes, with Δ*T*_m_ = ~-3°C compared to the native DNA, which is in the expected range. Both porphyrins display the characteristic absorption and emission spectra when measured either as single strand or as duplex with the unmodified complementary strands (Figure 2). The ground state absorbances in the mixed porphyrin duplexes **Z1** to **Z7** are largely unperturbed and can be described as a superposition of the absorbance of DNA duplexes which contain either **Zn-P** (**Z8**) or **2H-P** (**Z9**).

### Determining FRET Efficiency

**Figure 3:**
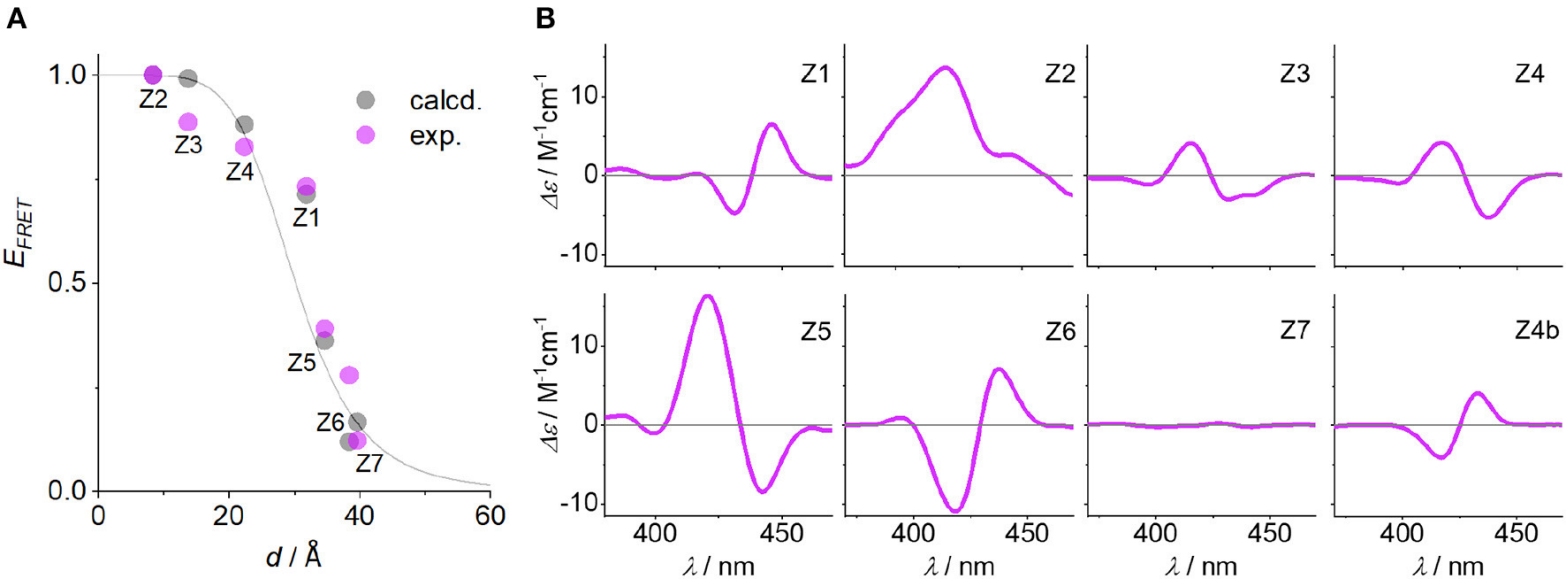
**(A)** *E*_*FRET*_ as function of **Zn**-**P** distance (*d*), comparing calculated (Preus et al., 2013) and measured FRET efficiencies (the gray line represents idealized behavior with κ^2^ = 2/3). **(B)** CD spectra of the **Zn**-**P** duplexes (porphyrin region), including the CD spectrum of **Z4b** where the angle of the porphyrin dipole moment has opposite sign to **Z4**. Conditions as in Figure 2.

The quantum yield and Förster distance of the **Zn-P**–**2H-P** pair were measured previously to be Φ = 0.12 and *R*_0_ = 28.4 Å, respectively (Burns et al., 2012). The distances and angles of the chromophores in the DNA duplexes used here were obtained from energy minimized structures, using the effective transition moment along the 5, 15-axis through the acetylene linker as determined by Berova et al. (Anderson, 1994; Matile et al., 1996; Huang et al., 2000). The calculated E_FRET_ values [see electronic supporting information [ESI] for details] (Preus et al., 2013) show deviation from the idealized model (κ^2^ = 2/3) (Figure 3A); for the calculation of the E_FRET_ efficiencies, κ^2^ was taken into account and calculated for each pair as described previously. The experimentally determined E_FRET_ values from the donor emission peak at 605 nm show a steady decrease in FRET efficiency with increasing donor-acceptor distance. As expected, **Z2** gives the highest E_FRET_ value due to the smallest porphyrin-porphyrin spacing. Conversely, **Z3** and **Z4** exhibit higher E_FRET_ values than **Z1**, even though they have the same base separation, or are separated by one extra base pair, respectively. These differences arise from the attachment points on the complementary strands which positions the porphyrins in **Z1** on opposite sides of the duplex, whereas in **Z2** and **Z3** the porphyrins are located in the same hemisphere of the duplex (see Figure 1 for DNA models).

The influence of the angle between the chromophores is clearly visible in the CD spectra. The excitonic coupling between the porphyrins display both positive and negative Cotton effects as a function of angle between the dipole moments (Figure 3B), which is defined as the projection angle of the dipole moments and is either (+) or (–) (viewing down the helical axis of the DNA). Generally, a positive Cotton effect at longer wavelengths and negative one at shorter wavelengths determines a positive exciton couplet and arises from a positive angle (positive exciton chirality) (Berova et al., 2009). This theory has successfully been used to determine the chirality in systems having identical chromophores, but not with mixed chromophore systems. Here, the angles match perfectly well with the CD chirality (Table 1), but only when assuming both porphyrins as equal. Thus, the porphyrins are close to being degenerate: the small ground state differences from zinc to free-base porphyrin are not discriminated by the exciton chirality method, and the system reports two identical chromophores. This is in contrast to the direct energy transfer as seen in FRET. To demonstrate this, we have synthesized the duplex **Z4b** where the relative position of the two porphyrins is inverted with respect to **Z4**, meaning that in **Z4b** the zinc porphyrin is now downstream of the free-base porphyrin. While the base pair separation and through-space distance of the porphyrins is maintained, the repositioning of the porphyrins results in an inverted exciton chirality, and the CD spectra are near mirror images of each other (Figure 3B; the E_FRET_ values were not determined for this system).

**Table 1:** Structural parameters, FRET efficiencies and CD data of the donor-acceptor systems.

**DNA duplex**	**Base pair separation**	**Porphyrin distance [Å] and dipole angle [deg]**	**E_**FRET**_, calcd. and exp. values**	**κ^2^ value**	**Exciton couplet $A_{\text{CD}}^{\text{a}}$ and exciton chirality^b^**
Z1	2	30.2/+158	0.71/0.73	2.704	+11.3/(+)
Z2	0	11.0/+22	1/1	1.001	+16.2/(+)
Z3	2	13.7/−60	0.99/0.89	0.759	−7.1/(–)
Z4	3	22.0/−92	0.88/0.83	0.963	−9.5/(–)
Z4b	3	21.6/+90	n.d.^c^	n.d.^c^	+8.2/(+)
Z5	5	34.7/−167	0.36/0.39	1.012	−24.8/(–)
Z6	7	39.8/+121	0.12/0.28	0.448	+18.0/(+)
Z7	11	41.1/+23	0.17/0.12	0.806	+0.4/(+)

a *The difference between the CD extrema at longer wavelengths and at shorter wavelengths (in Δε) determines the amplitude A_CD_ of the exciton couplet (Berova et al., 2009)*.

b *The exciton chirality corresponds to the sign of the dipole angle*.

c *not determined*.

### Detecting Ethidium Bromide Intercalation at Single Base Pair Level

We developed an intercalation assay to test the sensitivity of the porphyrin system. Given that the energy transfer method to measure distance changes is still ambiguous due to relatively small expected effects (e.g., see difference from **Z1** to **Z4**), the CD spectra seem to respond much more sensitively to the relative arrangement of the porphyrins. We therefore tested the response of the porphyrin arrays to the intercalation of ethidium bromide (EtBr). EtBr is a well-known intercalator by stacking inside the DNA duplex (sliding in-between base pairs) and is commonly used as a nucleic acid stain in gel electrophoresis to visualize the DNA bands. The binding of EtBr to DNA has been studied extensively, with reported global binding constants (*K*_D_) in the range of 3.4 × 10^3^ M^−1^ to 1.3 × 10^6^ M^−1^, depending mainly on salt concentration (Krugh et al., 1975; Scaria and Shafer, 1991; Vardevanyan et al., 2003; Alonso et al., 2006; Minasyan et al., 2006; Hayashi and Harada, 2007; Nafisi et al., 2007; Karacan and Okay, 2013). Also, the binding mode indicates that EtBr intercalates at approximately every two to three base pairs with n ~2.5 (Scaria and Shafer, 1991), though n-values ranging from 1.6 (Hayashi and Harada, 2007) up to 9 (Vardevanyan et al., 2003; Minasyan et al., 2006) have been reported. In addition, EtBr can show multiple binding interactions, including intercalation, semi-intercalation and electrostatic binding (Vardevanyan et al., 2003; Minasyan et al., 2006). We set out to test if our system would be suitable to directly detect the binding of an intercalator on both a large range and single base pair system.

**Figure 4:**
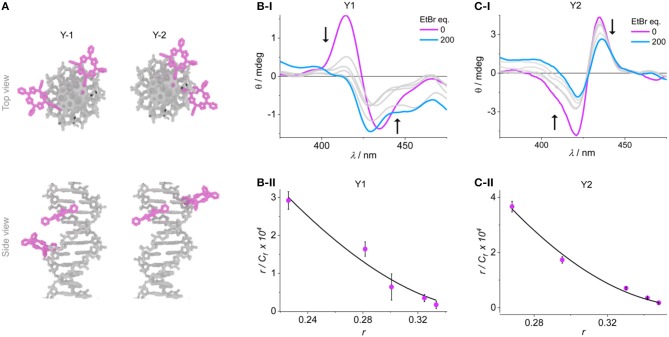
**(A)** Representation of the duplexes **Y1** and **Y2**. Example CD spectra of the titration experiments of **(B-i) Y1** and **(C-i) Y2**, the arrows indicate the peak shift upon addition of EtBr. Scatchard plots are shown in **(B-ii)** and **(C-ii)**, respectively, together with the fitted curves according to Equation (1).

We synthesized two additional porphyrin-DNA strands **Y1** and **Y2** (Figure 4), with two porphyrins being either at five base pairs apart, or on adjacent base pairs, respectively. This DNA system is shorter and simpler, providing only A-T base pairs in-between the porphyrin sites, and flanking G-C base pairs to maintain duplex stability. Since the metallation state does not influence the exciton coupling, both reporter porphyrins were used as 2H-porphyrins which also simplifies the synthesis by avoiding the post-synthetic metalation step. To probe the system, we titrated **Y1** with EtBr and observed a corresponding change in the exciton coupling of the porphyrins (Figure 4A and ESI). The large reduction in signal intensity can be attributed to a change in helicity of the DNA upon EtBr intercalation, and with it a change in chromophore distance and angle. The use of an excess of EtBr (>500 equivalents) did not indicate any significant changes in CD signal after addition of 200 equivalents, and up to that point the EtBr did not itself produce a significant signal in the porphyrin region that would interfere with the analysis. The binding constants were calculated from a non-linear fitting of the Scatchard plot of *r*/*C*_*f*_ vs. *r* (see Experimental section); the apparent global binding constant in our case is *K*_D_ = 1.43 ± 0.2 × 10^5^ M^−1^, with *n* = 2.6 binding sites. This tends toward the upper limit of binding constants reported. The analysis is based under the assumption that any outside binding, e.g., electrostatic interactions with the phosphate groups or groove binding, does not give rise to a change in porphyrin coupling as it would not lead to large structural changes. Also, we have not found any indication that EtBr would interact strongly with the porphyrins and lead to a change in CD signature (see ESI). Therefore, we can assume that this apparent *K*_D_ represents a value for the intercalation under our experimental conditions. The number of binding sites also compares well to reported values.

More interesting is the response of the system when the porphyrins are at adjacent base pairs as in **Y2**. The order of the porphyrins was switched to give opposite exciton chirality which distinguishes it clearly from the system **Y1**. The intercalation can again be monitored from a large reduction in the exciton coupling of the porphyrins. Here, it is assumed that any EtBr that would bind at random parts of the DNA (internally or externally) will not be detected, and only the single binding site occupation is observed. The data points were again fitted according to Equation (1) and yielded a *K*_D_ of 3.54 ± 0.4 × 10^5^ M^−1^, with *n* = 2.6. The *K*_D_ is higher than the global binding constant, but the weaker binding is a reflection of the competing sites outside of the porphyrin region which would lead to a lower effective molarity of the available EtBr. This is also shown by the same *n*-value for both systems. This result shows that the intercalation of a molecule can clearly be detected on the single base pair level using the strong exciton coupling of porphyrins, which is very sensitive down to the nanometer scale.

## Conclusions

Based on our previously established porphyrin-DNA system, we have demonstrated that varying the position of two porphyrins along a DNA helix can be used to create a molecular ruler, that responds well to changes in distance and structure. Using a well-established FRET pair based on zinc metalled and free-base porphyrins, the FRET efficiency can be correlated to the position of the porphyrins. However, the helicity can lead to ambiguity when FRET is used to determine the distance of the chromophores. This is mainly attributed to the helical structure and arrangement. Circular Dichroism, on the other hand, has revealed that the exciton coupling is independent on the metallation state, most likely because the absorbances of the two porphyrins (Zn, 2H) are very close. This is visible from the exciton couplet which follows the helical geometry of the porphyrin system, not the donor-acceptor energy order. Furthermore, the CD response is shown to be highly dependent on the relative orientation and distance of the porphyrins. This can be explored to monitor structural changes such as those that are induced by intercalation. While the system certainly needs to be evaluated in greater detail, the obtained binding constants for EtBr as a model system compare well with literature data. In this respect, changes can be detected down to the single base–pair level, which gives nanometer resolution in DNA analysis.

## Data Availability Statement

The datasets analyzed for this study can be found in the University of Southampton Institutional Research Repository, ePrints Soton: https://doi.org/10.5258/SOTON/D1227.

## Author Contributions

JB designed the ruler system. JB and JW synthesized the porphyrin-DNA strands and performed the experiments. JB, JW, and ES analyzed the data. ES supervised the project. ES and JB wrote the manuscript.

### Conflict of Interest

The authors declare that the research was conducted in the absence of any commercial or financial relationships that could be construed as a potential conflict of interest.
