# Supplementary material for: A Porphyrin-DNA Chiroptical Molecular Ruler With Base Pair Resolution
Source: Front Chem. 2020 Feb 26;8:113. doi: 10.3389/fchem.2020.00113 (PMC7054460; doi:10.3389/fchem.2020.00113)
Supplement: Supplementary file 1 [file Data_Sheet_1.pdf]

Supplementary Material

A porphyrin-DNA chiroptical molecular ruler with base pair resolution

Jonathan R. Burns[1*], James W. Wood[2], Eugen Stulz[2*]

[1]Department of Chemistry, University College London, Gower Street, London, WC1E 6B, UK.

[2]School of Chemistry & Institute for Life Sciences, University of Southampton, Highfield, Southampton SO17 1BJ, UK

*** Correspondence:**
Corresponding Author
jonathan.burns@ucl.ac.uk
est@soton.ac.uk

Table 1. DNA duplexes and sequences, where P is porphyrin dU modification. Zn metallated porphyrin is highlighted in red, free-base (2H) porphyrin in blue.

ID	Duplex sequences
Z1	3′-TAT TCA TAC AAC APT GAT TTC-5′<br>5′-ATA AGT ATG TTG TAA CPA AAG-3′
Z2	3′-TAT TCA TAC AAC APT GAT TTC-5′<br>5′-ATA AGT ATG TTG PAA CTA AAG-3′
Z3	3′-TAT TCA TAC AAC APT GAT TTC-5′<br>5′-ATA AGT ATG TPG TAA CTA AAG-3′
Z4	3′-TAT TCA TAC AAC APT GAT TTC-5′<br>5′-ATA AGT ATG PTG TAA CTA AAG-3′
Z4b	3′-TAT TCA PAC AAC ATT GAT TTC-5′<br>5′-ATA AGT ATG TPG TAA CTA AAG-3′
Z5	3′-TAT TCA TAC AAC APT GAT TTC-5′<br>5′-ATA AGT APG TTG TAA CTA AAG-3′
Z6	3′-TAT TCA TAC AAC APT GAT TTC-5′<br>5′-ATA AGP ATG TTG TAA CTA AAG-3′
Z7	3′-TAT TCA TAC AAC APT GAT TTC-5′<br>5′-APA AGT ATG TTG TAA CTA AAG-3′
Z8	3′-TAT TCA TAC AAC APT GAT TTC-5′<br>5′-ATA AGT ATG TTG TAA CTA AAG-3′
Z9	3′-TAT TCA TAC AAC ATT GAT TTC-5′<br>5′-ATA AGT ATG TTG TAA CPA AAG-3′
Y1	3′-GCG TAA TAP ACG-5′<br>5′-CGC APT ATA TGC-3′
Y2	3′-GCG TAA TAP ACG-5′<br>5′-CGC ATT ATA PGC-3′

Data obtained from the FRETmatrix calculation

The details for the calculation of the matrix can be found in ref [1].

Table 2. Specific data for the FRET system.

Modelled sequence:								
5'-A **X**AA G**X**A **XGX** **XGX** AAC **X**AA AG								
3'-T ATT CAT ACA ACA **Y**TG ATT TC								
	D strand	A strand	Separation	Donor pos.	Acc. Pos.	*R* /Å	κ^2	*E*	Porphyrin
	Z	2	0	14	13	8.4	1.001	1	1
	Z	3	2	14	11	13.8	0.759	0.991	0.886323
	Z	1	2	14	17	31.9	2.704	0.713	0.727523
	Z	4	3	14	10	22.4	0.963	0.881	0.827315
	Z	5	5	14	8	34.7	1.012	0.361	0.389964
	Z	6	7	14	6	38.5	0.448	0.118	0.278023
	Z	7	11	14	2	39.7	0.806	0.166	0.120958

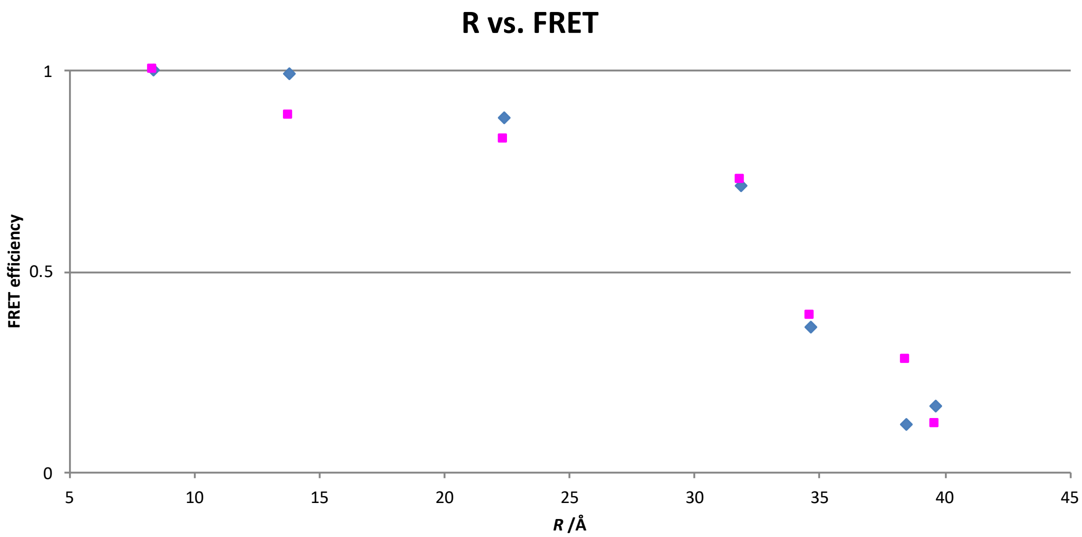

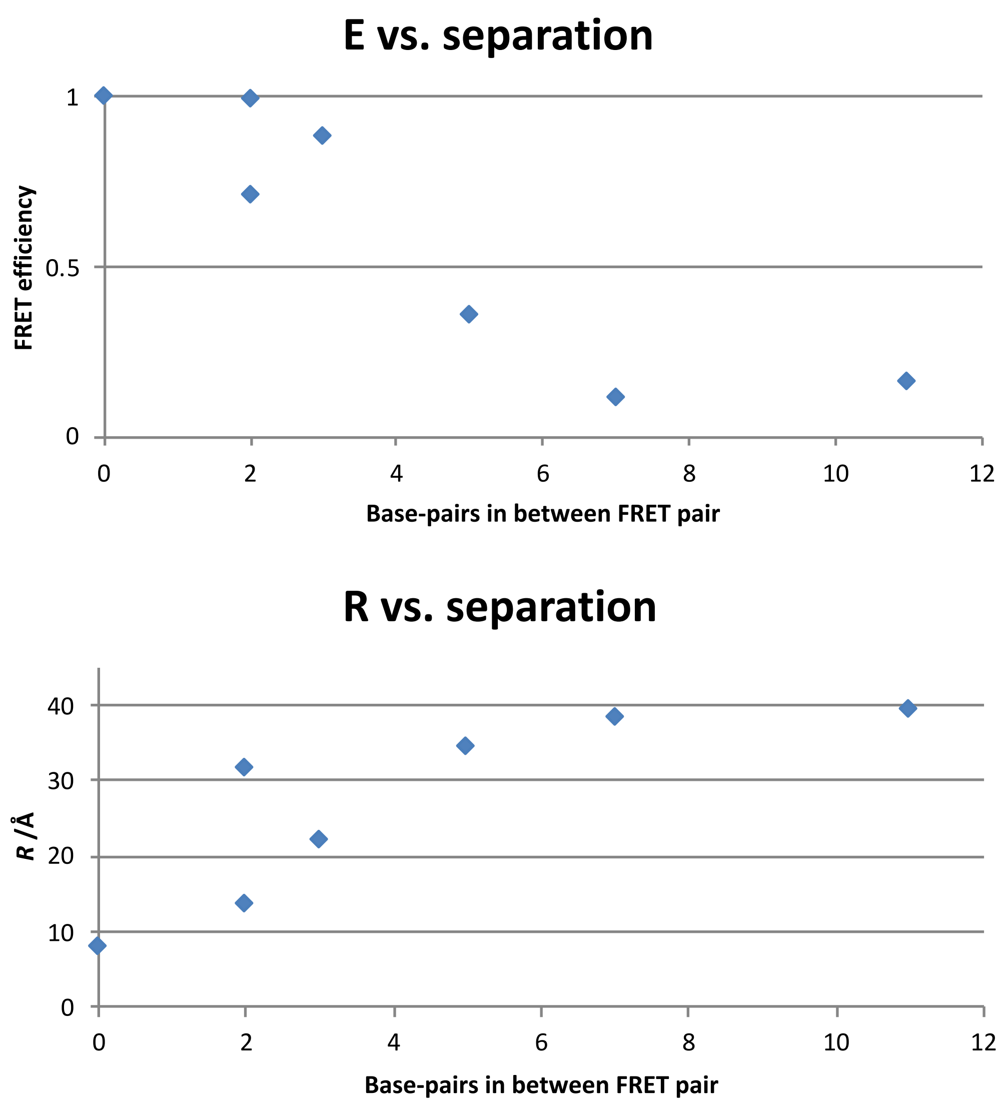

Supplementary Figure 1. The graphs show the plots of the table data indicating some deviation from the ideal FRET situation.

Titration experiments

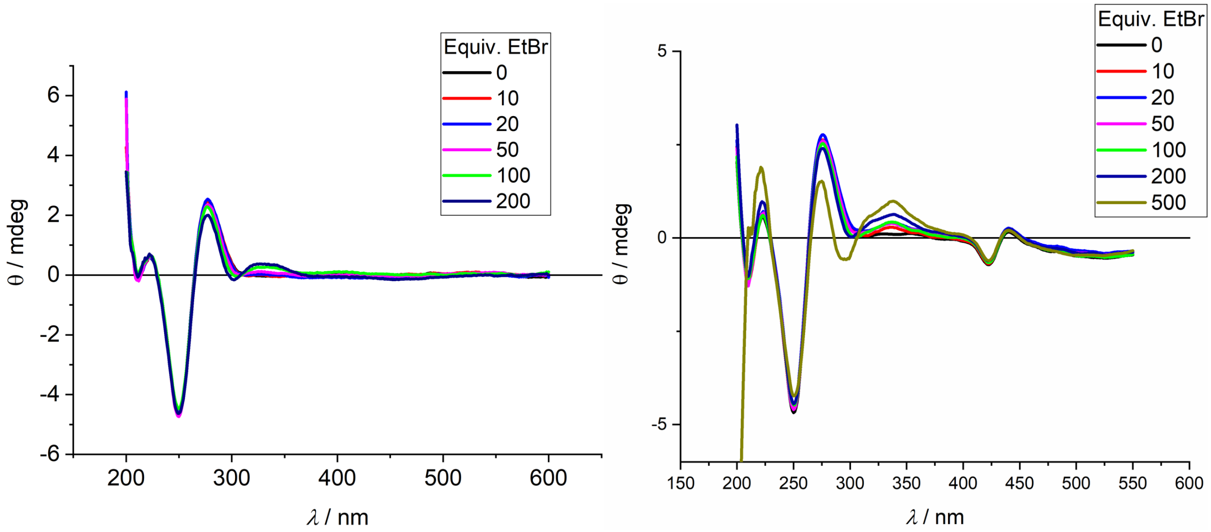

Supplementary Figure 2. Left: CD titration of unmodified DNA with EtBr. Right: CD titration of single porphyrin modified porphyrin-DNA with EtBr which shows no difference in the porphyrin region upon intercalation.

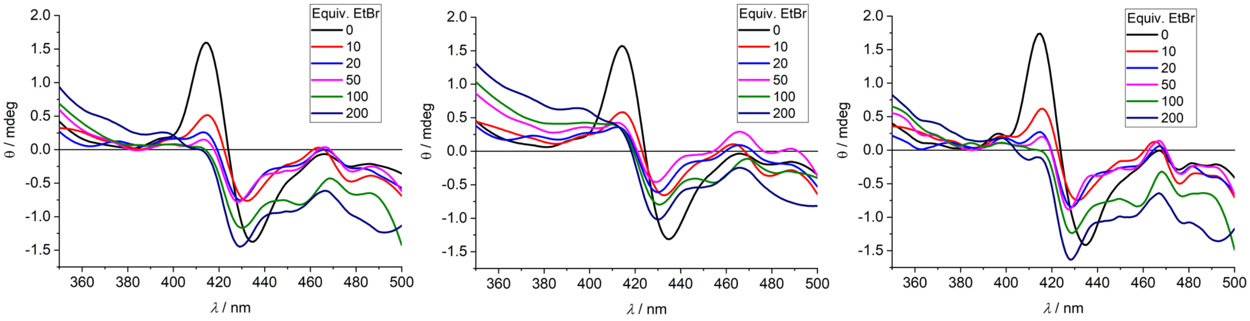

Supplementary Figure 3. CD titration of porphyrin-DNA system **Y1** with EtBr.

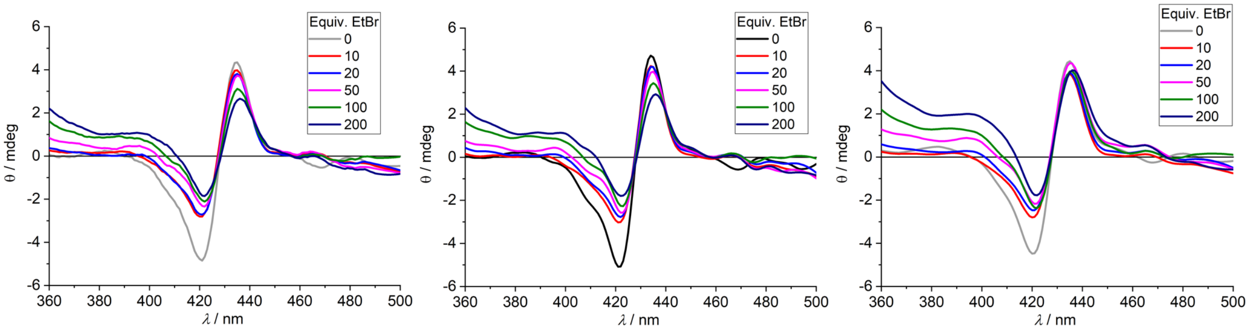

Supplementary Figure 4. CD titration of porphyrin-DNA system **Y2** with EtBr.

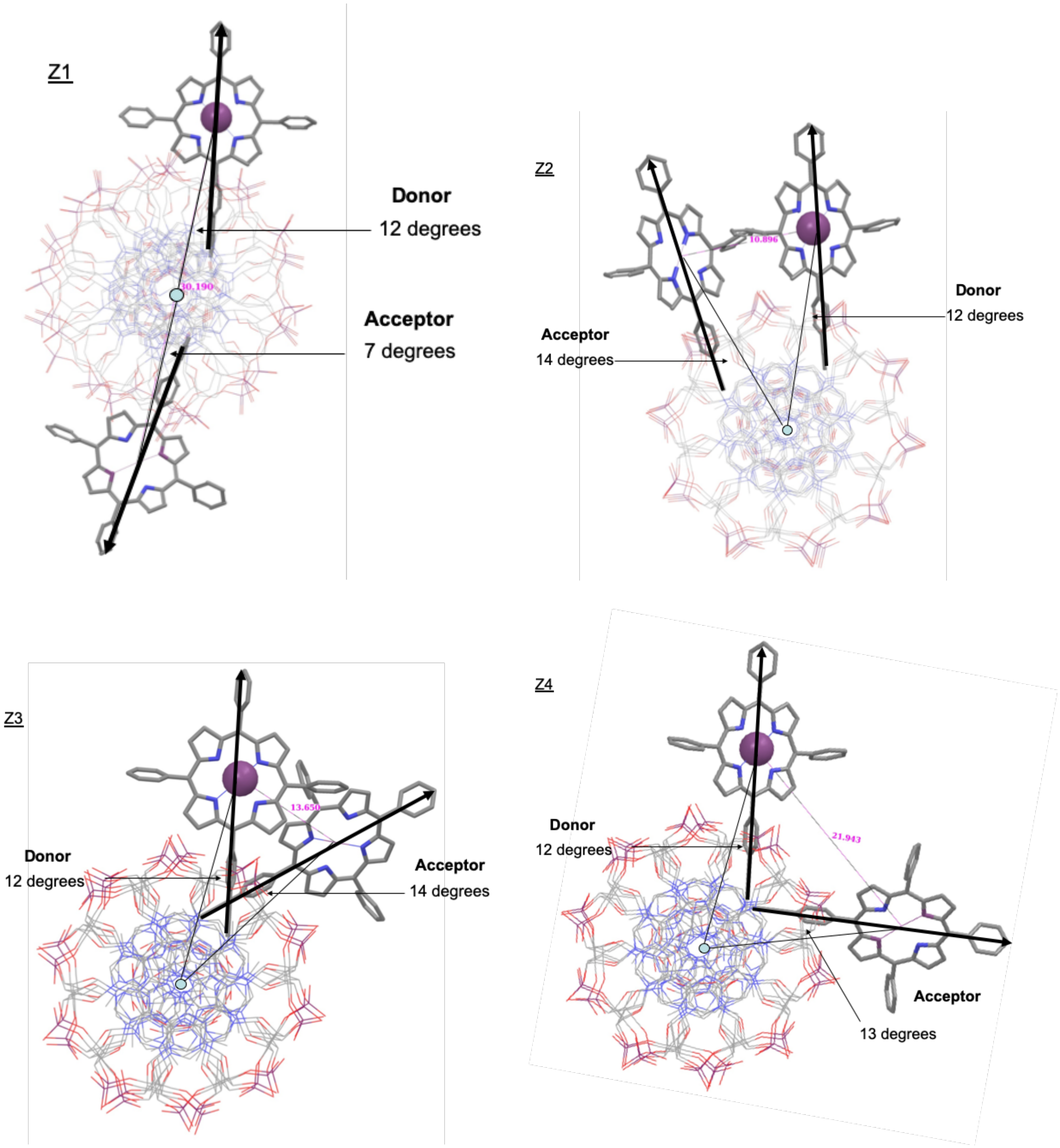

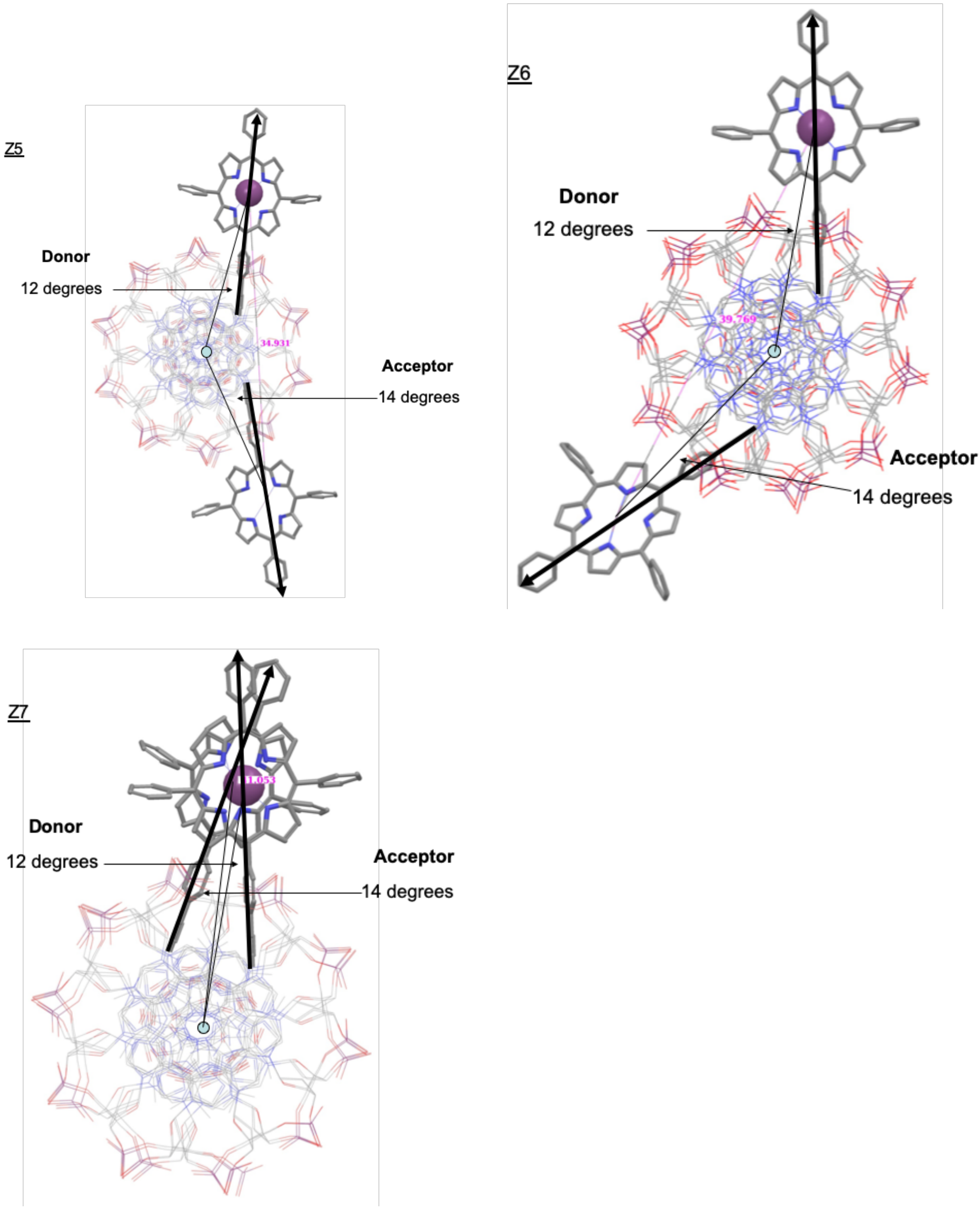

Supplementary Figure 5. Modelled structures of the porphyrin-DNA systems **Z1** to **Z7**.

References:

1. Preus, S., et al., *FRETmatrix: a general methodology for the simulation and analysis of FRET in nucleic acids.* Nucleic Acids Res, 2013. **41**(1): p. e18.
